# Combating infectious diseases of poverty: a year on

**DOI:** 10.1186/2049-9957-2-27

**Published:** 2013-11-18

**Authors:** Shang Xia, Pascale Allotey, Daniel D Reidpath, Pin Yang, Hui-Feng Sheng, Xiao-Nong Zhou

**Affiliations:** 1 National Institute of Parasitic Diseases, Chinese Center for Disease Control and Prevention (China CDC), Shanghai 200025, People’s Republic of China; 2 WHO Collaborating Centre for Malaria, Schistosomiasis and Filariasis, Key Laboratory of Parasite & Vector Biology, Ministry of Health, Shanghai 200025, People’s Republic of China; 3 Jeffrey Cheah School of Medicine and Health Sciences, Monash University Sunway Campus, Kuala Lumpur, Malaysia

**Keywords:** Infectious diseases of poverty, Health system, Surveillance and response systems, Coinfection, Syndemics

## Abstract

The *Infectious Diseases of Poverty* journal, launched a year ago, is a platform to engage outside the traditional disciplinary boundaries, and disseminate high quality science towards the improvement of health. This paper reviews the milestone achievements during its first year of operation. The journal has filled an important niche, addressing some of the main priorities in the *Global Report for Research on Infectious Diseases of Poverty*. Highlights include the publication of three thematic issues on health systems, surveillance and response systems, as well as co-infection and syndemics. The thematic issues have foregrounded the importance and innovation that can be achieved through transdisciplinary research. The journal has been indexed by PubMed since April 2013, with the publication of a total of 38 articles. Finally, the journal is delivering to wider range readers both in developing and developed countries with sustained efforts with a focus on relevant and strategic information towards elimination of infectious diseases of poverty.

## Multilingual abstract

Please see Additional file 1 for translation of the abstract into the six official working languages of the United Nations.

## Background

Infectious diseases are inextricably linked to populations living in the poor conditions [1,2]. This remains the case with emerging infections and with the re-emergence of infectious diseases for which prevention and cures have been successful in other populations [3,4]. Challenges persist with the dynamic evolution of our ecology and the disease agents, including pathogens, vectors, as well as biological, social, cultural, political and environmental factors. Although there are rapid technological and scientific developments that propose solutions to some aspects of diseases of poverty, current paradigms in research still struggle with the balance of integration of the knowledge produced by the growing numbers of disciplines that are able to contribute to our understanding of the complexity in health and disease [5-7]. Furthermore, mechanisms for the sharing and translation of knowledge into policies and effective practice needs to be given greater priority, particularly to improve access to low and middle income countries that have the highest endemicity [8,9].

*Infectious Diseases of Poverty* (IDP), http://www.idpjournal.com, was launched on 25 October 2012 at the *2nd Global Symposium on Health Systems Research* held in Beijing, with the specific purpose to foster interdisciplinary and transdisciplinary research that explicitly highlights the intersection of poverty and other ecological factors with disease. The IDP journal provides a forum for researchers to engage outside the traditional disciplinary boundaries and for policy makers to find and disseminate high quality science towards the improvement of the health of vulnerable populations. The journal encourages publications that aim:

(i) to identify and assess the research base underlying important current and future public health options, choices and decisions;

(ii) to highlight information gaps and research gaps;

(iii) to review a wide range of topic areas, methods and strategies in diseases of poverty; and

(iv) to facilitate a much needed dialogue between policy makers, public health practitioners, control staff and academic researchers and their donors.

The IDP journal builds on knowledge translation activities initiated by the UNICEF/UNDP/World Bank/World Health Organization Special Programme for Research and Training in Tropical Diseases (TDR), especially within the TropIKA.net initiative. In November 2011, the National Institute of Parasitic Diseases (NIPD) of Shanghai, People’s Republic of China, hosted a *Consultation on setting up an international Partnership for TropIKA.net* as the initiative was being phased out by TDR. The IDP journal was one of the three priority areas identified by the participants and NIPD took the lead in establishing the journal in collaboration with the open access publisher, BioMed Central (BMC) [10].

**Figure 1:**
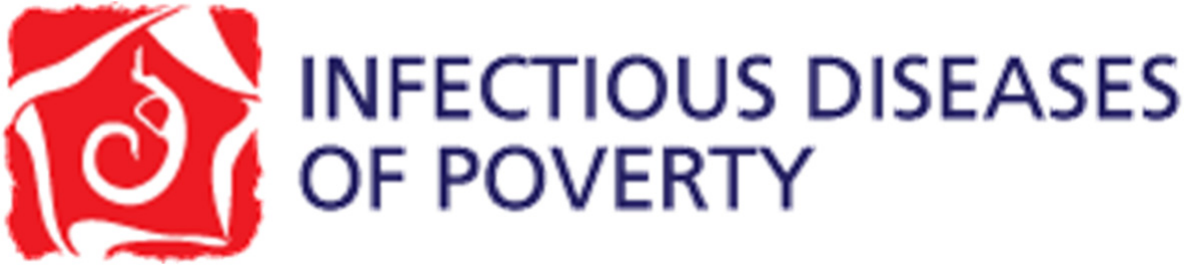
The logo of Infectious Diseases of Poverty.

The IDP Journal takes on the unique mission "One health - One world" put forward by the *Global Report for Research on Infectious Diseases of Poverty* (Global Report) [1]. The mission of the journal is expressed in its logo (Figure 1), which consists of three components: (i) the worm (schistosoma) represents infectious diseases; (ii) the distorted house around the worm represents poverty and environmental challenges; (iii) the whole image is akin to an outdated wax seal, which indicates a persistent problem in need of innovative solutions. This editorial summarizes the progress of the journal after one year of publication.

## Issues addressed

### The breadth and depth of issues covered

**Figure 2:**
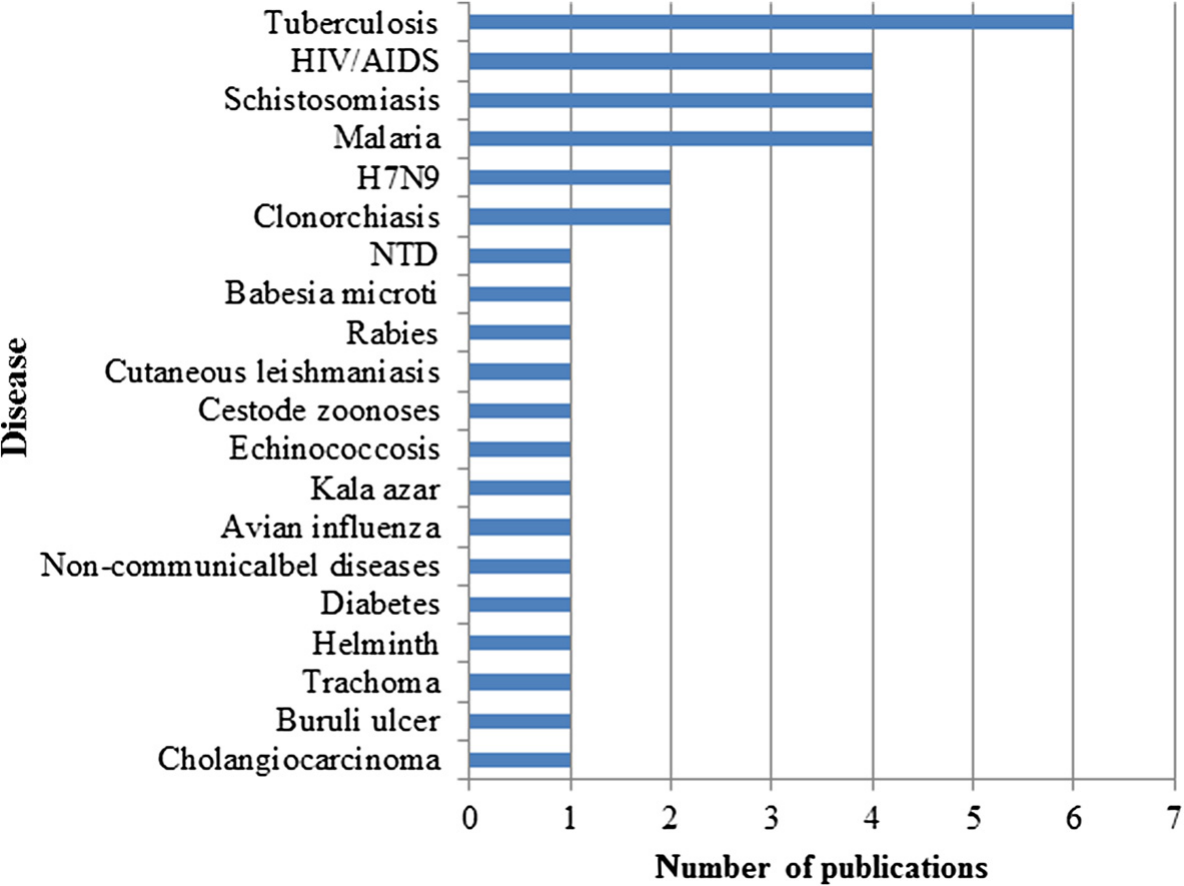
The number of publications by diseases.

A total of 15 diseases were covered by the 38 free online and open access articles, ranging from TB, HIV/AIDS, schistosomiasis, malaria, H7N9 influenza, and clonorchiasis to co-infections with non-communicable diseases and with pregnancy. Several neglected tropical diseases were also addressed such as babesiasis, leishmaniasis, echinococcosis, helminth infections, avian influenza, rabies, trachoma, buruli ulcer, diabetes, and cholangicarcinoma, etc. (Figure 2).

Three thematic issues were published: (i) Health Systems Research for Infectious Diseases of Poverty; (ii) Surveillance and Response to Infectious Diseases of Poverty; and (iii) Co-infection and Syndemics. All of them covered transdisciplinary research, ecohealth approaches, and translational sciences to combat the infectious diseases of poverty.

In the first thematic issue entitled “Health Systems Research for Infectious Diseases of Poverty”, the main purpose of the collection was to review the recent progress on the roles of the health systems in combating the infectious diseases of poverty in order to call for more research on health systems [11,12]. The 12 articles emphasized the essential characteristics of poverty and provided evidence on technical solutions to managing the infectious diseases that afflict poor populations world-wide [13,14]. The articles discussed treatments, indicating the importance of pharmaceuticals for neglected diseases, as well as delivery strategies to reach impoverished populations, and highlighted the lack of research on (i) innovative programs that provide diagnostics and treatment for infectious diseases to hard-to-reach rural and urban communities, (ii) exploring research on other health system components, and (iii) broadening the evidence base to increase understanding of effective and sustainable interventions to reduce the burden of infectious disease among the poor.

The collection of papers on “Surveillance and Response to Infectious Diseases of Poverty”, published 15 articles from traditional neglected tropical diseases to the newly outbreaks of H7N9 flu that make international impact significantly [13,15]. Based on the innovative and effective "One health - One world" paradigm, six different research priorities were recommended, including (i) dynamic mapping of transmission; (ii) capture of population dynamics; (iii) modeling based on a minimal and essential database approach; (iv) implementation of mobile-health (m-health) and sensitive diagnostics; (v) design of response packages tailored to different transmission settings and levels; and (vi) validation of approaches and responses, all of which were outcomes of the *First Forum on Surveillance Response System Leading to Tropical Diseases Elimination,* which was held in Shanghai in June 2012 [13].

In the third thematic issue, entitled “Co-infection and Syndemics”, a total of six articles were published on co-infections or syndemics between HIV/AIDS, tuberculosis, malaria, and other neglected tropical diseases, such as intestinal parasitic infections, schistosomiasis, babesiasis, clonorchiasis, and diabetes, etc. [16-19]. In fact, co-infection is of particular human health importance because pathogenic agents can interact within the host, while syndemics refers to the aggregation of two or more diseases in a population, that can be expanded further to situate the occurrence of multiple diseases within the context of poverty and other mitigating factors that support and perpetuate poor health. Given that social conditions can contribute to the clustering, form and progression of a disease at both individual and population levels, it is a great challenge to understand the processes that generate these patterns of co-infection and syndemics.

All three thematic issues have trigged more research on exploring innovative and transdisciplinary approaches, which could be applied in the resource limited settings or in the field of better medical and public health interventions in the poor population.

### Achieving the agenda for action proposed by the Global Report

In face of challenges in infectious diseases of poverty for control programmes, the Global Report emphasized the need to break the vicious cycle between poverty and infectious diseases, and to forge an escape for the poor and vulnerable population through the uptake of global health research solutions [1]. A total of five options for action were put forward by the Global Report as high level priorities [20,21].

Among the 38 articles in the first two issues of *Infectious Diseases of Poverty*, some articles were responding to those five options for action as advocated in the Global Report [1].

The option for action 1 (see Table 1), for instance, “Create and use a new index of infectious diseases of poverty to serve as a surrogate marker of national socioeconomic development” calls for doing research on defining new indices based on current prevalence of infectious diseases to inform policy maker on where resources are needed, which control effort are to be put in place, when should intervention be implemented, where the research activities be prioritized, and how to use them to evaluate and monitor the control programmes, etc. During the past year, four articles reported on disease burden by measuring impact of diseases at local, national and global levels [12,15,19,22], three articles described how to measure the ability of the health systems to predict, prevent and deal with disease outbreaks and to deliver effective interventions [11,12,14]; two articles dealt with the government commitment to tackle the problems on infectious diseases of poverty [23,24]; and three articles focused on socioeconomic factors to measure determinants of health [17,25,26].

**Table 1:** **Five actions to break the vicious cycle of poverty and infectious diseases (**from Global Report [1]**)**

**Options**	**Description of actions**
Option for action 1	Create and use a new index of infectious diseases of poverty to server as a surrogate marker of national socioeconomic development
Option for action 2	Implement a "One health - One world" strategy in relation to research for infectious diseases of poverty
Option for action 3	Actively promote research ownership with enabling policies by disease endemic countries
Option for action 4	Create an innovation platform to foster a culture of innovation to benefit public health
Option for action 5	Create an online global platform of research resources to inform on strategies, policies and funding commitments

The option for action 2 calls for policy-makers, funders and academic community to embrace a "One health - One world" strategy, in particular, to foster essential multidisciplinary and multisectoral approaches for a full continuum of research. In this regard, four researchers had linked with governments of disease endemic countries that develop intersectoral frameworks and encourage cooperation in the field of zoonosis [19,27-29], and additional four investigations had highlighted the collaboration across various ministries as well as among medical and veterinary doctors [13,22,30,31].

In the option for action 3, four articles had worked on developing research congruent with the burden of infectious diseases of poverty in their own populations [26,32-34], three articles demonstrated an increase in research activities and improved research leadership [14,30,31], three articles were trying to develop regional partnerships aiming for building research infrastructure, human resources and research capacity [22,32,35], and two articles focused on how to create policies and develop plans to guide international investments towards the identified research priorities [13,36]. However, there is still a significant need to increase national investment for research and the translation of research to strategies for health [31,35].

Unfortunately, to date, there has not yet been any publications related to the option for action 4 and the option for action 5. We hope the readers are able to promote this kind of research, and enhance this field with the development of the journal. It is expected that the global platform would be populated by information from multiple sources and provide a database for research on infectious diseases of poverty [4,37]. In particular we would strongly encourage and promote more research or review articles to be published on the issues of 10 reasons to do research on infectious diseases of poverty and research solutions for global health, which were raised by the Global Report (Table 2).

**Table 2:** **Ten reasons to do research on infectious diseases of poverty (from Global Report**[1]**)**

**No.**	**Reasons**	**Detail description**
1	Break the vicious cycle of poverty and infectious diseases	The interrelationships between health, infectious diseases and poverty are dynamic and complex. Timely, targeted research will prevent infectious diseases from driving more people into poverty
2	Forge an escape for the poor and vulnerable	Poor people living in the areas most affected by environmental factors are least able to respond to the challenges of environmental and climate change. Interactive, interdisciplinary research can identify ways to mitigate risk factors, establish the potential impact of interventions on the environment and direct future interventions to minimize risk
3	Tackle multiple problems	Research will help understand both causes and consequences of polyparasitism, coinfection and comorbidities with non-communicable diseases on people, societies and systems. An integrated understanding of the complex relationships underpins effective integrated health system delivery and effective disease control programmes
4	Commute the life sentence	Many people must live with the long-term debilitating effects of past or current infection. Research can find ways to mitigate the consequences of chronic and persistent lifelong infection and its secondary complications and associated stigma
5	Be prepared-forewarned is forearmed	Surveillance is essential at all levels to understand patterns of emergence, including the spread of drug and insecticide resistance. Mapping, monitoring and evaluation of these trends are critical. Access to such surveillance data allows us to anticipate and respond to emergent, re-emergent and drug-resistant diseases
6	Reach the hardest to reach	By identifying ways to strengthen health infrastructure and better deliver services in impoverished areas, we can reach disenfranchised populations who continue to struggle with the burden of poverty and disease. Health systems research can create positive synergies between disease control and wider health systems in poor regions
7	Prevent loss in translation	Progress along the route from basic research to clinical and public health practice is slow and patchy. Integrated multidisciplinary research programmes should aim to anticipate and avoid potholes along the route to the introduction of more effective interventions
8	Identify small changes that can make a big difference	Relatively low levels of investment in evidence-based interventions can have a big impact. Small modifications in where and how we deliver treatments and care can achieve dramatic improvements. Effective research that demonstrates positive effects from small modifications should be rapidly scaled up in poor communities
9	Stay focused on the light at the end of the tunnel	Much has been achieved to date and even the most difficult situations are not irreversible. Significant progress will continue to be made if investment in coordinated research programmes is expanded and sustained
10	Act quickly on what we know	Policy-makers and global funders need to have access to the right information at the right time to inform decisions that draw on the evidence of what works, and feed “best buys” into health policy, health budgets and the operations of health systems. Research data must therefore be rapidly translated into effective tools for policy-makers

It is interesting to note that the top five highly accessed articles reached more than 3,000 visits for each article, and up to 10,000 visits of the top one. Among the top five articles, three articles are related to the transdisciplinary approaches with targeting cross-cutting issues, such as environmental changes, social determinants and health system [27,38,39]. Another two articles put forward the research priorities based on the gap analysis between control programmes and research capacity, in the field of malaria, schistosomiasis, and echinococcosis [24,34].

### Success in editorial process

In the first two inaugural volumes from October 2012 to October 2013, IDP journal attracted strong submissions and developed a readership within the BMC model, including (i) open-access for readers, (ii) high quality of published work, and (iii) a professional and transparent editorial process.

**Figure 3:**
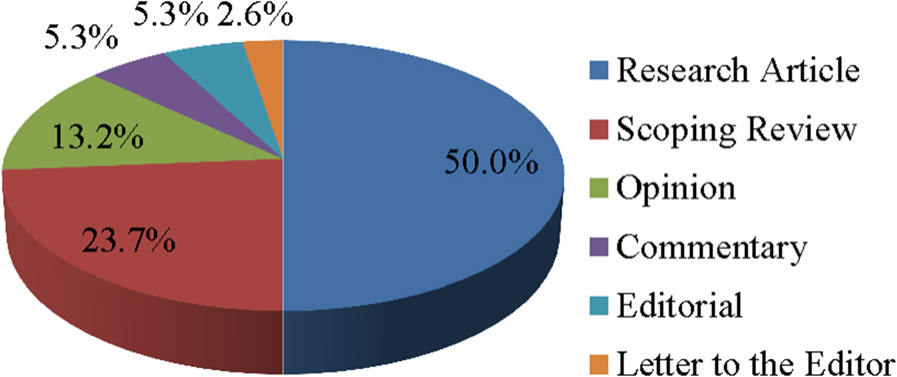
The percentage of different types of articles published during the first year.

**Figure 4:**
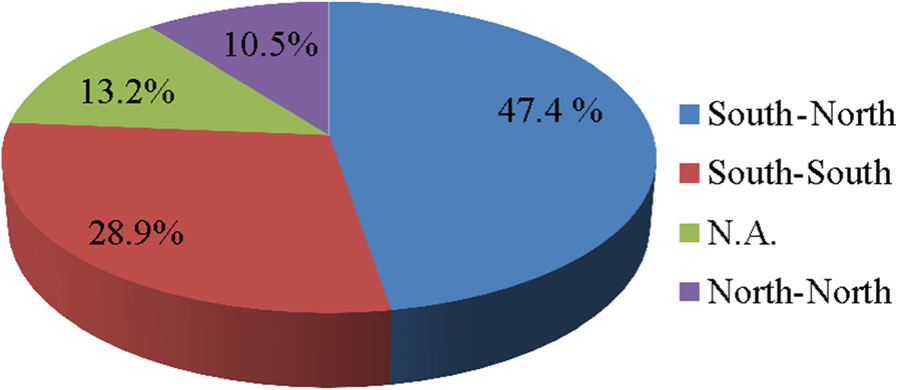
The percentage of each cooperation model (N.A. means single institution involved in among 38 articles published).

The open-access model allows wide penetration and dissemination into low income settings. A total of 38 publications have been online in the first and second volumes of IDP journal in the first year, with the 6 types of articles including research articles accounting for 50.0% of total publications, followed by scoping reviews (23.7%), opinion pieces (13.2%), commentaries (5.3%), editorials (5.3%), and letters to the editor (2.6%) (Figure 3). A total of 145 authors or co-authors were presented in 38 publications, whose geographic distribution showed were mainly from Asia (53.1%) and Africa (24.1%), followed by Europe (13.1%), North America (7.6%), Oceania (1.4%) and Lain America (0.7%). About 3.2 institutions in average involved in each articles, which mainly cooperated through south–north model accounting for 47.4%, followed by south-south cooperation (28.9%), and north-north cooperation (10.5%) (Figure 4). Therefore, the south-south cooperation in the field of infectious diseases of poverty needs to be further strengthened (Additional file 2).

Scientists from 39 countries have submitted their manuscripts to this journal, in which Africa and Asia accounting for 39% and 37% of the total number of submitted manuscripts, respectively, followed by Latin America (10%), Oceania (5%), North America (4%), and Europe (4%). While the visits came from the whole of the world, and the top ten countries for visiting the journal are ordered by following countries: United States, China, India, Nigeria, Australia, United Kingdom, Philippines, Pakistan, South Africa, which indicating 70% of visits from low and middle income countries (Additional file 2).

**Figure 5:**
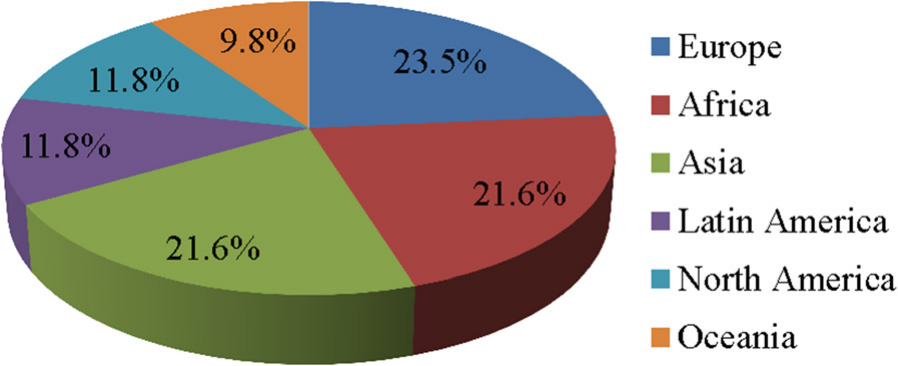
Distribution of editor board members by geographic regions.

Secondly, the high quality of publications has been demonstrated in the first year. For example, some of publications have been cited more than 3 times from the Thomson Reuters database and more than 5 times from the Google Scholar database during less than one year. The journal has also maintained a high level of rigor in the peer review process. Approximately 35% of submission currently passed the peer review process. Manuscripts accepted were from Europe (40%) and North America (40%), followed by Asia (38%), Africa (20%), and Oceania (17%). This kind of publication quality was contributed by an excellent mix of experts from disease-endemic countries as well as leading research institutions in high-income countries, with a total of 51 editorial board members. Most of editorial board members had experiences serving the think tank in WHO/TDR for the Global Report, they are selected from Europe (23.5%), Africa (21.6%), Asia (21.6%), Latin America (11.8%), North America (11.8%), and Oceania (9.8%) (Figure 5). There is a good gender balance and several yong scientists have joined the board (Additional file 2).

Thirdly, professional editorial process has increased the efficiency in production of all publications. Two special efforts were made by the editorial team. One is that the abstract of each publication was translated into 6 UN working languages. The other one is the short time of efficient peer review for the decision and publication, with average time was about 65 days and 25 days, respectively. Those efforts have been apprased by authors widely. A strong commitment by the editoiral office in China to this work is demonstrated by the waiving of article processing costs in the initial stage of the new journal until the end of 2014, and this is to ensure that researchers who have innovative ideas for transdisciplinary research are able to disseminate their ideas based on the quality of the science and without being hindered by cost restrictions.

## Conclusion

The journal fills an important niche, in line with the high-level Global Report put forth by the World Health Organization in 2012 [40]. The journal was indexed by PubMed in April 2013, and the next logical step is to obtain an official impact factor through ISI Web of Knowledge. The journal has already established itself as an important outlet and holds promise of becoming a leading and influential journal in the field of global health and infectious diseases in future years. The data from co-authors’ institutions indicated the south-south cooperation need to be further strengthen in the future. We look forward to delivering on this promise to our acknowledged authors and wide readers with sustained efforts by professional editorial team. It is expecting more readers to publish their research or review articles on the issues of 10 reasons to do research on infectious diseases of poverty and research solutions for global health, proposed by the Global Report [1].

## Competing interests

The authors declare that they have no competing interests.

## Authors’ contributions

SX, PA, DR, XNZ drafted the first version of the manuscript. SX, PY, HFS, XNZ perform the data analysis. SX, PA, DR, XNZ participated in the revisions. All authors read and approved the final manuscript.

## Supplementary Material

Additional file 1 Multilingual abstracts in the six official working languages of the United Nations. Click here for file

Additional file 2 Success in editorial management with evidences of detail data. Click here for file
