# Supplementary material for: Combating infectious diseases of poverty: a year on
Source: Infect Dis Poverty. 2013 Nov 18;2:27. doi: 10.1186/2049-9957-2-27 (PMC3892074; doi:10.1186/2049-9957-2-27)
Supplement: Additional file 1 — Multilingual abstracts in the six official working languages of the United Nations. [file 2049-9957-2-27-S1.pdf]

Tanslation of the abstract into the six official working languages of the United Nations

مكافحة الأمراض المعدية الناجمة عن الفقر: على مدى عام

شانغ شيا، باسكال ألوتي، دانيال ريدباث، بينغ يانغ، هوي-فنغ شين وشياو نونغ تشو

ملخص

تمثل صحيفة ŭ ŭ ŭ FγÜŭFΘ ŭ ، الصادر أول أعدادها في العام الماضي، منصة لتجاوز حدود التخصصات التقليدية، ونشر العلوم عالية الكفاءة، وذلك لتعزيز المستوى الصحي. ويستعرض هذا التقرير الإنجازات الأساسية خلال العام الأول لممارسة الصحيفة لنشاطها. احتلت الصحيفة مكانة هامة بتناولها لبعض الأولويات الرئيسية في Ķŭ Üŭ Ч̆ ŭķ ŭ ŭ FγÜŭFΘ ŭ . وتضمنت المواضيع الرئيسية ثلاث قضايا موضوعية عن الأنظمة الصحية وأنظمة المراقبة والتصدي والعدوى المرافقة والأمراض المزدوجة. أبرزت القضايا الموضوعية مدى أهمية الابتكارات التي يمكن تحقيقها من خلال البحوث متعددة التخصصات. وقد قام موقع النشرات الطبية PubMed بإدراج الصحيفة في منشوراته منذ أبريل 2013 بإجمالي عدد منشورات تبلغ 38 مقالاً. وأخيراً نجد أن الجهود المتواصلة تعمل على نشر الصحيفة بين عدد كبير من القراء في الدول النامية والمتقدمة، مع التركيز على المعلومات الاستراتيجية ذات الصلة، نحو القضاء على الأمراض المعدية الناجمة عن الفقر.

Translated from English version into Arabic by Ran Abdel Rahman, through

TRANSLATORS
WITHOUT BORDERS

防治贫困所致传染病——“贫困所致传染病”杂志创刊一周年

Shang Xia, Pascale Allotey, Daniel Reidpath, Ping Yang, Hui-Feng Shen, et Xiao-Nong Zhou

摘要

一年前出版的“贫困所致传染病”杂志，是一个提升卫生水平而超越传统学科、传播高质量科学知识的平台。本文对该杂志在第一年中所取得的成就进行了综述。该杂志填补了重要的空白，着重关注“贫困所致传染病研究全球报告”中提出的重点领域。主要是出版了卫生系统、监测响应系统、多重感染与复合流行等 3 个专辑，这些专辑内容涉及到了重要的、创新性的交叉学科研究前沿。该杂志已出版了 38 篇文章，并在 2013 年 4 月被 PubMed 数据库引用。杂志所提供的迈向消除贫困所致传染病相关的策略信息，正在吸引着包括发展中国家和发达国家的读者群。

Translated from English version into Chinese by Xiao-Nong Zhou.

La lutte contre les maladies infectieuses liées à la pauvreté : un an après

Shang Xia, Pascale Allotey, Daniel Reidpath, Ping Yang, Hui-Feng Shen, et Xiao-Nong Zhou

Résumé

La revue *Infectious Diseases of Poverty,* lancée il y a un an, est une tribune visant à dépasser le cloisonnement traditionnel des disciplines et à disséminer un savoir scientifique de qualité en vue de promouvoir la santé. Cet article fait le bilan des réalisations importantes accomplies durant la première année d'existence de la revue. Celle-ci comble un créneau important, abordant certaines des priorités décrites dans le *Global Report for Research on Infectious Diseases of Poverty* (rapport mondial concernant la recherche sur les maladies infectieuses liées à la pauvreté). Parmi les plus notables de ces réalisations, on trouve la publication de trois numéros thématiques : sur les systèmes de soins de santé, sur les systèmes de surveillance et d'intervention, et sur la coinfection et les syndémies. Ces numéros thématiques ont mis en avant les innovations qui peuvent être réalisées par le biais de la recherche transdisciplinaire, ainsi que l'importance de ce type de recherche. Depuis avril 2013, la revue est répertoriée par PubMed, qui affiche un total de 38 articles publiés. Enfin, la revue atteint des lecteurs d'un plus large éventail de disciplines, tant dans les pays en développement que dans les pays développés, concentrant ses efforts sur la diffusion d'information pertinente et stratégique visant l'élimination des maladies infectieuses liées à la pauvreté.

Translated from English version into French by Anne Duranceau, through

TRANSLATORS
WITHOUT BORDERS

Борьба с инфекционными заболеваниями, вызванными бедностью: текущий год

Шанг Ксиа, Паскаль Оллотей, Даниэль Рейдпат, Пинг Янг, Хуй-Фенг Шен и Ксиао-Нонг Джоу

Резюме

Запущенный год назад журнал *«Инфекционные заболевания, вызванные бедностью»* является платформой, выходящей за пределы традиционных дисциплинарных границ, и направляет высокотехнологичную науку на улучшение состояния здоровья населения. В данной работе дается обзор основных достижений журнала в течение первого года работы. Журнал заполнил собой важную нишу, выделив ряд основных приоритетов в *«Глобальном отчете об исследовании инфекционных заболеваний, вызванных бедностью»*. Прежде всего, стоит отметить публикацию трех тематических статьей о системах здравоохранения, контроля и реагирования, а также параллельном инфицировании и синдемии. Тематические статьи подчеркнули всю важность проблемы и те инновационные решения, которые могут быть реализованы посредством междисциплинарного исследования. С апреля 2013 года журнал включен в список PubMed за публикацию 38 статей. Наконец, журнал обращается к широкому кругу читателей, ориентируясь на актуальную и стратегически важную информацию, касающуюся искоренению инфекционных заболеваний, вызванных бедностью.

Translated from English version into Russian by Irina Zayonchkovskaya, through

TRANSLATORS
WITHOUT BORDERS

Combatiendo las enfermedades infecciosas asociadas a la pobreza: un año en marcha

Shang Xia, Pascale Allotey, Daniel Reidpath, Ping Yang, Hui-Feng Shen, y Xiao-Nong Zhou

Síntesis

La revista *Infectious Diseases of Poverty*, lanzada un año atrás, es un programa comprometido con la divulgación de manera no tradicional de conocimientos científicos de alta calidad para la mejora de la salud. Este documento analiza los logros más significativos durante su primer año de operación. La revista respondió a una importante demanda, cubriendo algunas de las principales prioridades destacadas en el *Global Report for Research on Infectious Diseases of Poverty*.

Los principales logros incluyen la publicación de tres ediciones sobre el tema sistemas de salud, sistemas de vigilancia, coinfección y sindemia. Dichas ediciones temáticas destacaron en primer plano las posibilidades de innovación que pueden obtenerse a través de la investigación transdisciplinaria. La revista ha sido registrada por PubMed desde abril de 2013, con un total de 38 artículos publicados. Por último, la publicación alcanza a un amplio rango de lectores tanto en países desarrollados como en vías de desarrollo, con esfuerzos sostenidos destinados a brindar información estratégica y de importancia para la eliminación de las enfermedades infecciosas asociadas a la pobreza.

Translated from English version into Spanish by FerVilamajo, through

TRANSLATORS
WITHOUT BORDERS
