# Supplementary material for: Combating infectious diseases of poverty: a year on
Source: Infect Dis Poverty. 2013 Nov 18;2:27. doi: 10.1186/2049-9957-2-27 (PMC3892074; doi:10.1186/2049-9957-2-27)
Supplement: Additional file 2 — Success in editorial management with evidences of detail data. [file 2049-9957-2-27-S2.pdf]

Additional File 2.

Success in editorial management with evidences of detail data

In the first two inaugural issues from the October 2012 to the October 2013, *Infectious Diseases of Poverty* has managed to attract strong submissions and developed a readership, which can be reflected in the following three aspects.

1. Open-access model accepted widely

1.1 Types of articles

Its open-access model allows wide penetration and dissemination into low income settings. A total 38 articles have been published online in the first and second volumes of *Infectious Diseases of Poverty* between 25 October 2012 and 24 October 2013, with 6 types of articles: research articles accounting for 50.0% of the total publications, followed by scoping reviews (23.7%), opinion pieces (13.2%), commentaries (5.3%), editorials (5.3%), and letters to the editor (2.6%).

All research reports presented data from the original researches or the field investigation outcomes in poor settings. Most of them were field surveys on prevalence and disease burden of relevant diseases in poor settings, such as malaria, schistosomiasis, filariasis, Kala Azar, clonorchiasis, buruliasis, babesiasis, trachoma, tuberculosis, HIV/AIDS, bird (avian) influenza. Importantly, the results presented new findings on risk factors, vulnerable population, and changes in impact, as well as investigations on new and user-friendly tools for intervention or diagnosis, new approaches to delivering the intervention in existing health systems and modeling methodology for alerting or making predictions of new outbreaks.

In the scoping reviews, publications provided an authoritative review on a precise question. Specifically, they highlighted the available research data that may have the potential to lead to innovation and knowledge application in public health, the recommended information from the gap analysis that may prevent progress towards effective intervention. Particularly interesting topics are those information resulted from the multidisciplinary analysis or reviewing, such as (i) comparison study on the outcomes from the tropical diseases control programmes, different health systems and innovative research between Africa and Asia continents, (ii) the network modeling providing new scenarios on epidemiology of tropical diseases, (iii) research gaps and

challenges in control or elimination of infectious diseases of poverty, (iv) the disease burden and impact factors on zoonosis by investigations in integration with medical and veterinary medicine, (v) co-morbidity between infectious diseases and non-communicable diseases.

In the opinions articles, one opinion related to innovation technology in infectious diseases of the developing world, two opinions focused on the importance of health system in the control programmes of the tropical diseases, and another two highlighted the importance some of neglected tropical diseases which will lead to the chronic diseases or non-communicable diseases consequently.

One commentary and one letter to the editor extended previously published research including additional controls and confirmatory results, which provided research priorities in application of new approaches leading to the elimination of infectious diseases of poverty.

1.2 The submission and publications

The submission and publications from 2012 to 2013 came from a total of 39 and 16 countries, respectively (Table S1). The number of submission was led by China, followed by Nigeria, Australia, India, Tanzania, Cameroon, Ethiopia, Mexico, USA, Nepal, Brazil, Thailand, UK, Ghana, Hong Kong, Egypt, Uganda, Canada, Mali, Zambia, Pakistan, Yemen, Sri Lanka, Argentina, Benin, Japan, Mozambique, Bangladesh, Iran, Malaysia, Morocco, Congo, Portugal, Peru, Sudan, Philippines, and Sweden. The number of publication was led by China, Ghana, Nepal, Hong Kong, USA, and Tanzania, followed by Nigeria, Ethiopia, Thailand, UK, Mali, Zambia, Australia, Philippines, and Sweden.

Table S1. The number of publication and submission by each country

Country	Publication		Submission		Acceptance rate	
	2013	2012	2013	2012	2013	2012
China	7	5	11	11	63.6%	45.5%
Nigeria	2	0	8	7	25.0%	0.0%
Cameroon	0	0	5	0	0.0%	0.0%
Ethiopia	1	0	4	1	25.0%	0.0%
Nepal	2	0	4	0	50.0%	0.0%
Mexico	0	0	4	1	0.0%	0.0%
Thailand	1	0	3	0	33.3%	0.0%
Australia	0	1	3	3	0.0%	33.3%
Canada	0	0	3	0	0.0%	0.0%
Brazil	0	0	3	1	0.0%	0.0%

USA	1	2	3	2	33.3%	100.0%
India	0	0	2	4	0.0%	0.0%
Uganda	0	0	2	0	0.0%	0.0%
Egypt	0	0	2	0	0.0%	0.0%
UK	1	0	2	1	50.0%	0.0%
Pakistan	0	0	1	0	0.0%	0.0%
Mali	1	0	1	0	100.0%	0.0%
Tanzania	1	2	1	5	100.0%	40.0%
Mozambique	0	0	1	0	0.0%	0.0%
Switzerland	1	0	1	0	100.0%	0.0%
Bangladesh	0	0	1	0	0.0%	0.0%
Sudan	0	0	1	1	0.0%	0.0%
Yemen	0	0	1	0	0.0%	0.0%
Japan	0	0	1	0	0.0%	0.0%
Kenya	0	0	1	1	0.0%	0.0%
Hong Kong	1	1	1	1	100.0%	100.0%
Benin	0	0	1	0	0.0%	0.0%
Morocco	0	0	1	0	0.0%	0.0%
Argentina	0	0	1	0	0.0%	0.0%
Iran	0	0	1	0	0.0%	0.0%
Zambia	1	0	1	0	100.0%	0.0%
Ghana	1	2	1	2	100.0%	100.0%
Malaysia	0	0	1	0	0.0%	0.0%
Sri Lanka	0	0	1	0	0.0%	0.0%
Portugal	0	0	0	1	0.0%	0.0%
Peru	0	0	0	1	0.0%	0.0%
Sweden	0	1	0	1	0.0%	100.0%
Philippines	0	1	0	1	0.0%	100.0%
Congo	0	0	0	1	0.0%	0.0%
Total	21	15	78	46		

1.3 Access of articles

Based on the custom dashboard from Google analytics, the visits came from the whole of the world, and the top ten countries for visiting the journal are ordered by following countries: United States, China, India, Nigeria, Australia, United Kingdom, Philippines, Pakistan, South Africa, which indicating 70% of visits from low and middle income countries (LMICs) (Table S2).

The article access numbers are growing but will require some effort on dissemination. The new visits account for 67.1% of the total visits (Figure S1, S2).

Table S2. Visits by top ten countries (up to 30 September, 2013)

Country/Territory	Visits	Pages /visit	% new visits
China	1459	6.69	33.72%
United States	1211	2.18	82.16%
United Kingdom	479	2.39	78.29%
India	395	2.98	81.77%
Brazil	333	2.99	76.88%
Japan	276	4.37	23.91%
Australia	216	2.97	68.98%
Tanzania	200	2.76	51.50%
Nigeria	198	3.09	66.67%
(not set)	193	2.04	72.02%

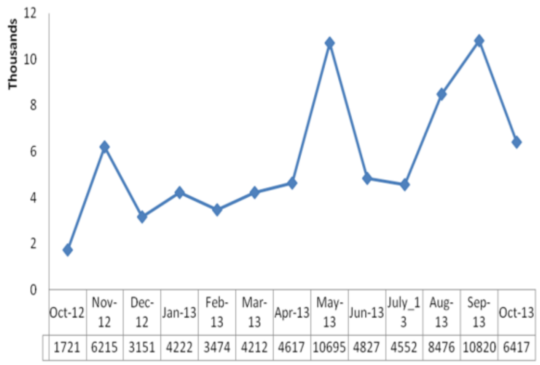

Figure S1. The trend curve indicating the number of article accesses from October 2012 to October 2013.

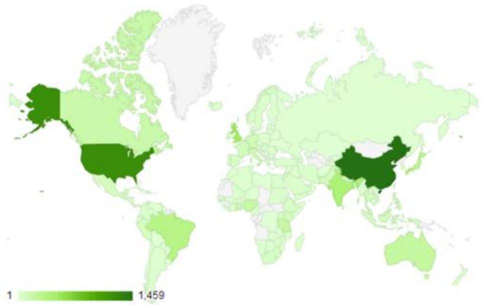

Figure S2. The geographic distribution of the visits, showing visits coming from whole of the world.

1.4 Global distribution of authors or co-authors

A total of 145 authors or co-authors were presented in 38 publications, whose geographic distribution showed were mainly from Asia (53.1%) and Africa (24.1%), followed by Europe (13.1%), North America (7.6%), Oceania (1.4%), and Lain America (0.7%) (Figure S3). The top number of authors or co-authors was from China, followed by USA, UK, Nigeria, Swaziland, Tanzania, Hong Kong, Ghana, Mali, India, Nepal, Ethiopia, Zambia, Uganda, Thailand, Australia, Sweden, Philippines, Norway, Kenya, Japan, France, Brazil, and Benin, etc. (Figure S4).

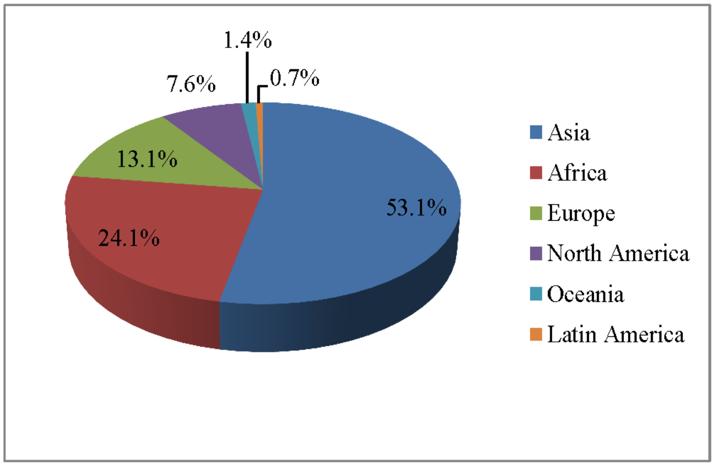

Figure S3. The geographic distribution of authors and co-authors among 38 publications.

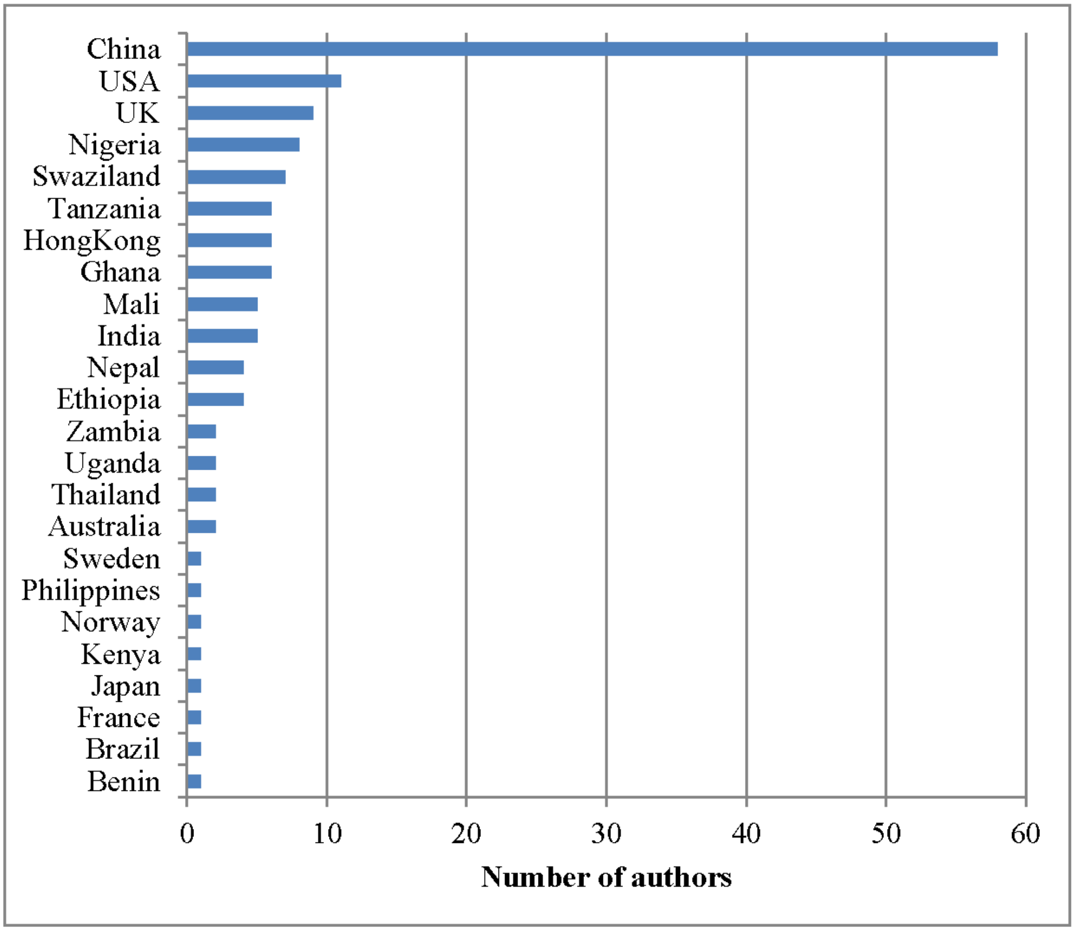

Figure S4. The number of authors and co-authors among 38 publications by countries.

1.5 Strong cooperation among institutions both from south and north

The average number of co-authors for each article was 4.34, ranged from 1 to 13 (Figure S5). It is showed that the average number of institutions involved in each articles was 3.2 institutions, ranged from 1 to 11 institutions, which showing a good collaboration in those research (Figure S6). Those researches were mainly cooperated by south-north cooperation accounting for 47.4%, followed by south-south cooperation (28.9%), and north-north cooperation (10.5%).

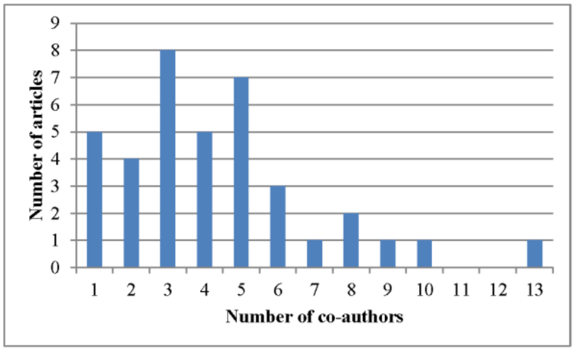

Figure S5. The number of co-authors per article.

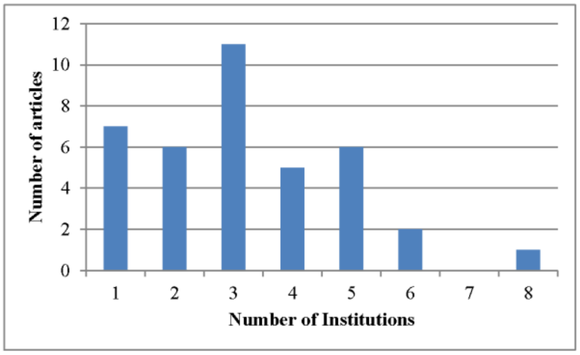

Figure S6. The number of institutions involved in each articles.

2. High quality of published work

2.1 The citation numbers of articles published have been increased with the time.

Some of publications have been cited more than 3 times from from the Thomas Routes database and 5 times from Google Scholar database during less than one year. The detail citation showed in the Figure S7. Table S3 summarizes the characteristics and number of citations of the 10 most highly cited papers published in the first year of *Infectious Diseases of Poverty*.

Table S3. Top ten publication in terms of citing articles

First Author	Article	Year	No. citation
Bergquist, Robert	Control of neglected tropical diseases in Asia Pacific: implications for health information priorities.	2012	1
Butler, Colin D	Infectious disease emergence and global change: thinking systemically in a shrinking world.	2012	1
Huntington, Dale	Health systems perspectives - infectious diseases of poverty.	2012	1

Qian, Men-Bao	Time to tackle clonorchiasis in China.	2013	2
Qian, Men-Bao	The global epidemiology of clonorchiasis and its relation with cholangiocarcinoma.	2012	3
So, Anthony D	Technology innovation for infectious diseases in the developing world.	2012	1
Tambo, Ernest	Scaling up impact of malaria control programmes: a tale of events in Sub-Saharan Africa and People's Republic of China.	2012	1
Wei, Xiaolin	Providing financial incentives to rural-to-urban tuberculosis migrants in Shanghai: an intervention study.	2012	2
Zhou, Xiao-Nong	Elimination of tropical disease through surveillance and response.	2013	3
Zhou, Xiao-Nong	Prioritizing research for "One health - One world".	2012	3

* Cited publications: 11,   number of citation: 19 (based on from the Thomas Routes database)

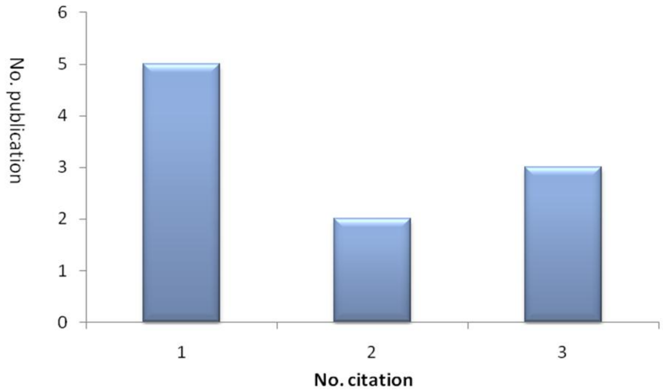

Figure S7. The number of citation for publications (from the Thomas Routes database).

2.2 Attracted submissions from whole of the world.

When taking look the geographic distribution of authors, the submission of manuscripts was led by Africa and Asia accounting for 39% and 37% of the total number of submitted manuscripts, respectively, followed by Latin America (10%), Oceania (5%), North America (4%), and Europe (4%) (Figure S8a). The publication number was also led by Africa and Asia accounting for 30% and 53% respectively, followed by Europe and North America, as well as Oceania and Latin America (Figure S8b).

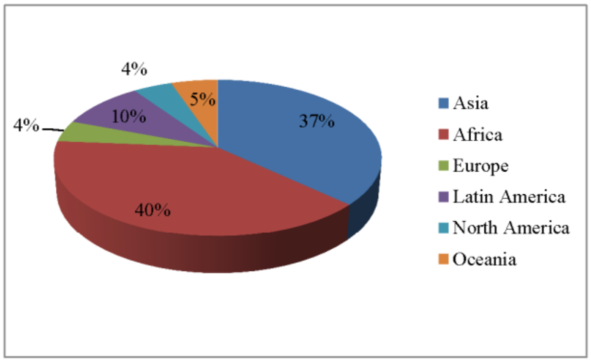

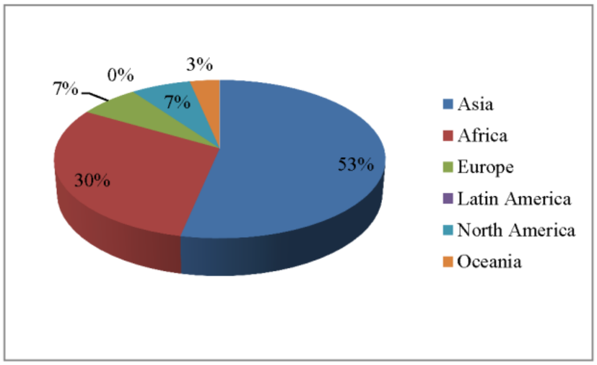

Figure S8. The ratios of articles by geographical region (a. the ratios of submission by geographical region, b. the ratios of publication by geographical region)

2.3 High quality in peer review.

The journal has maintained a high level of rigor in the peer review process. This means that the acceptance rate is on par with most of the top tier public health journals. Approximately 35% of submission currently pass the peer review process. Manuscripts accepted were from Europe (40%) and North America (40%) followed by Asia (38%), Africa (20%), and Oceania (17%) (Figure S9).

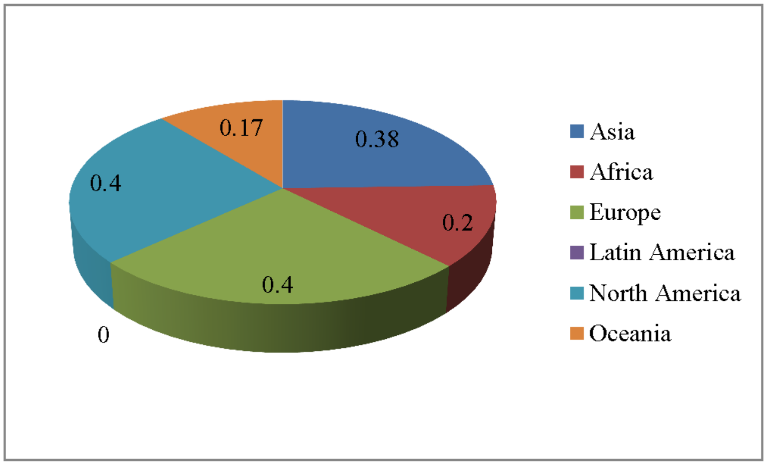

Figure S9. Acceptance rates by geographical region

3. Highly professional editorial process

3.1 The journal has an outstanding editorial board.

An excellent mix of experts from disease-endemic countries as well as leading research institutions in high-income countries was composed with a total of 51 editorial board members currently. Most of editorial members are selected from Europe (23.5%), Africa (21.6%) and

Asia(21.6%), followed by Latin America (11.8%), North America(11.8%), and Oceania (9.8%) (Figure S12). Most of editorial board member had experiences serving the think tank for the WHO/TDR for the Global Report on Research for the Infectious Diseases of Poverty. There is a good gender balance and several yong scientists have joined the board.

Secondly, abstracts of each publication are translated into 6 UN working languages, which enhances accessibility and dissemination for whom from developing countries. The data showed that the access rate for the abstract was led by English (US) language accounting for 62.6% of the total access numbers.

Thirdly, the short time for the decision and publication, with average time was about 65 days and 25 days, respectively. It is also can see that the time to first decision is becoming shorter with the time (Figure S10).

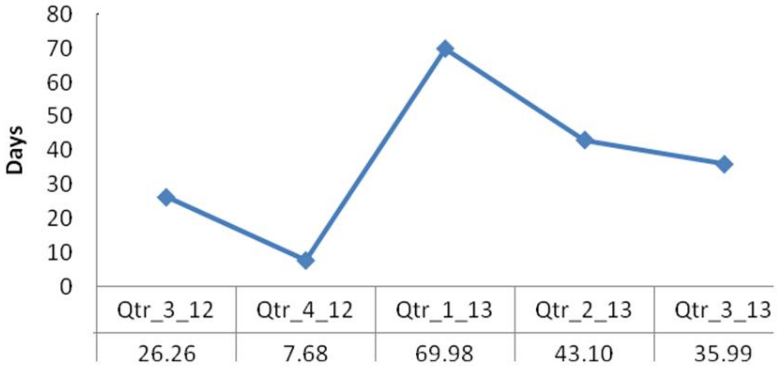

Figure S10. Time to first decision after peer review.
